# Genome-wide association study reveals major loci for resistance to septoria tritici blotch in a Tunisian durum wheat collection

**DOI:** 10.1371/journal.pone.0310390

**Published:** 2025-02-06

**Authors:** Maroua Ouaja, Bikash Ghimire, Bochra Amina Bahri, Medini Maher, Sahbi Ferjaoui, Sripada Udupa, Sonia Hamza

**Affiliations:** 1 Laboratory of Cereal Breeding, Institut National Agronomique de Tunisie, University of Carthage, Tunis, Tunisia; 2 Department of Plant Pathology, Institute of Plant Breeding, Genetics and Genomics, University of Georgia, Griffin, GA, United States of America; 3 Banque Nationale des Gènes, Boulevard du Leader Yasser Arafat Z. I Charguia 1, Tunis, Tunisia; 4 Centre Régional des Recherches en Grandes Cultures, Beja, Tunisia; 5 International Center for Agricultural Research in the Dry Areas (ICARDA), Rabat, Morocco; Indian Agricultural Research Institute, INDIA

## Abstract

Septoria tritici blotch (STB) is a devastating fungal disease affecting durum and bread wheat worldwide. Tunisian durum wheat landraces are reported to be valuable genetic resources for resistance to STB and should prominently be deployed in breeding programs to develop new varieties resistant to STB disease. In this study, a collection of 367 old durum and 6 modern wheat genotypes previously assessed using single Tunisian *Zymoseptoria tritici* isolate TUN06 during 2016 and 2017 and TM220 isolate during 2017 were phenotyped for resistance to a mixture of isolates (BULK) under field conditions. Significant correlations for disease traits using the three different inoculums were observed. Using 7638 SNP markers, fifty-one marker-trait associations (MTAs) for STB resistance were identified by genome-wide association study (GWAS) at Bonferroni correction threshold of -log_10_(*P*) > 5.184 with phenotypic variance explained (PVE) reaching up to 58%. A total of eleven QTL were identified using TUN06 isolate mean disease scoring (TUNMeanD and TUNMeanA) including threeQTL controlling resistance to both isolates TUN06 and TM220. A major QTL was identified on each of chromosomes 1B, 4B, 5A, and 7B, respectively. The QTL on 7B chromosome colocalized with *Stb8* identified in bread wheat. Four QTL including the major QTL identified on chromosome 1B were considered as novel. SNP linked to the significant QTL have the potential to be used in marker-assisted selection for breeding for resistance to STB.

## Introduction

Durum wheat (*Triticum turgidum* L. subsp. durum (Desf.) Husn.) is a staple crop that represents the third most important cereal crop worldwide [1] and is mainly grown under rain-fed conditions of the Mediterranean areas. The characterization of durum wheat landraces collected from the Mediterranean basin revealed a higher genetic diversity compared to other regions of the world [2]. Therefore, this region was considered as a center of diversification and production of durum wheat [3, 4]. Within the Mediterranean region, Tunisia is one of the main centers of diversity of durum wheat [5, 6]. The molecular and morphological characterization of old Tunisian durum wheat cultivars showed a high level of genetic diversity [7–10]. Hence local durum wheat landraces have been grown and selected under combined natural and anthropic pressures in a low-input agriculture system, developing specific adaptation and multiple resistances to local pathogen strains [2, 11–14].

The foliar blotch disease caused by the hemibiotroph fungus *Zymoseptoria tritici* (*Z*. *tritici*) (Desm.) Quaedvlieg & Crous (previously known as *Mycosphaerella graminicola* (Fuckel) J. Schort.in Cohn), is a major threat to wheat production that might cause up to 40% yield losses under the conducive conditions of north Tunisia [15–17]. Despite *Z*. *tritici* infects durum and bread wheat, a physiological specialization of this pathogen to either bread or durum wheat has been observed [18, 19] and warrants a proper understanding of *Z*. *tritici*-durum wheat interaction for effective breeding of *Z*. *tritici* resistance in durum wheat [20]. Hence the co-evolution of durum wheat and *Z*. *tritici* in the Mediterranean basin has contributed to rapid adaptation of *Z*. *tritici* isolates, due to large scale cultivation of durum wheat. Hence, virulence specificity depend on the cultivar but also on the host specie, bread wheat or durum wheat the strain have been isolated [21]. Recent studies have demonstrated that the old landraces of Tunisian durum wheat harbor effective sources for resistance to virulent Tunisian *Z*. *tritici* isolates [15, 17, 22, 23]. Therefore, the characterization of Tunisian durum wheat landraces using virulent isolates from this region would greatly contribute to enhance breeding for resistance to STB in durum wheat.

Both qualitative and quantitative resistances have been reported for resistance to STB in wheat. Qualitative resistance is usually controlled by major genes that confer complete resistance and follow the gene-for-gene model [24]. Quantitative resistance is controlled by many minor genes and widely reported in wheat cultivars at both seedling and adult growth stages [25–27]. The genetic architecture of resistance to STB in bread wheat has been mostly evaluated in bi-parental populations by detecting quantitative trait loci (QTL) [26, 28–33]. Recently, 25 QTL associated with STB resistance were identified on 16 chromosomes using a multi-parental winter bread wheat population [34]. To date, twenty-three STB genes and over 160 QTL for resistance *to Z*. *tritici* have been identified in bread wheat [24, 35, 36]. The recent availability of high-definition genotyping platforms [37] divulged new perspectives for exploring untapped genetic material and precise identification of genomic locations associated with STB resistance. Dense markers have been applied in genome-wide association studies (GWAS) to identify genetic involved in STB resistance in diverse bread wheat populations, in field or growth chamber environments [36, 38–45]. However, only minor QTL for STB resistance with less than 17% of the phenotypic variation (PVE) was detected in these studies. In durum wheat, a GWAS was conducted on a panel of Ethiopian landraces after natural STB infection in field conditions and has identified five putative QTL explaining no more than 10% of the PVE [40]. Since Ethiopian durum germplasm is genetically distant from the durum wheat gene pool commonly used in the Mediterranean basin [14, 46], the characterization of North African durum wheat germplasm needs to be investigated. In addition, North Africa is considered a secondary center of origin of durum wheat, and it is suggested that the co-evolution of durum wheat and *Z*. *tritici* in this region has created novel and adapted pathogen strains [47, 48]. Therefore, GWAS on durum wheat genotypes using isolates from this region is likely required to add new resistance sources to the durum wheat gene pool, which can be used for gene pyramiding [22, 23, 49].

In a previous study, a population structure analysis on a diversity collection of 373 durum wheat accessions and cultivars, genotyped with 286 SNPs and phenotyped in the field for STB resistance using two isolates TUN06 and TM220 during two consecutive seasons (2016 and 2017) and 2017, respectively showed that STB resistance originated from East Mediterranean area [50]. In this study, the same collection was phenotyped with a bulk of isolates (BULK) in 2018 and used in addition to the previous results of TUN06 and TM220 isolates to conduct a GWAS to assess the stability of the QTL across different inoculum. Inoculation with BULK could reveal new QTL different from the ones identified in single isolate inoculations.

## Materials and methods

### Wheat materials

A set of 367 durum wheat accessions and six modern cultivars was used in this study. Accessions were collected from four regions in Central (the Sahel and Kairoun) and southern (Gabes and Medenine) Tunisia [50]. These accessions described in a supplementary file (S1 and S2 Tables) l were deposited in National Gene Bank of Tunisia GRIN-Global repository (*http*:*//tn-grin*.*nat*.*tn*) according to their NGBTUN ID *under the following web link* http://www.tn-grin.nat.tn/gringlobal/search.

### Field trials and inoculums

A panel of 373 durum wheat cultivars was assessed in the field of CRRGC Beja. The panel was assessed for its resistance to STB. Inoculum of TUN06 isolate was used in 2016 and 2017 whereas TM220 isolate was used only in the 2017 field experiment [50]. A bulk inoculum (BULK) provided by the Septoria Platform in Tunisia was used for the 2018 field experiment and consisted of a mixture of five durum wheat- derived isolates (TNSP16381, TNSP16394, TNSP16418, TNSP16448, and TNSP16453) collected from the CRRGC Beja in 2016. The isolates were mixed after isolation and monosporal culture to make the BULK. Field trials were set up as reported by Ouaja et al. (2023) [50] and followed an Augmented Randomized Complete Block Design (ARCBD), including six blocks spaced apart at 1 m. Each block consisted of 70 accessions and seven checks. Blocks were 1 m width linearly drilled and accessions were sown 20 cm spaced apart. The checks included six susceptible modern cultivars ‘Karim’, ‘Khiar’, ‘Om Rabia’, ‘Salim’, ‘Maali’ and ‘Nasr’ and a resistant accession ‘Agili39’ [49].

### Inoculation and screening for resistance to *Zymoseptoria tritici*

The inoculation was conducted with the TUN06 isolate during 2016 and 2017, TM220 isolate during 2017 and BULK inoculum during 2018 growing season. The BULK inoculum was prepared according to the procedure described by Ouaja et al. (2023) [50]. Spores were adjusted to 10^6^ spore/ml in distilled water and 0.1% Tween20. Accessions in all experimental plots were inoculated twice with a motor sprayer, at the three-leaf (approximately GS21 Zadok’s scale) and stem elongation stage (approximately GS37) as described by Ouaja et al. (2023) [50].

BULK disease severities were assessed at 15, 37, and 54-days post the second inoculation (dpi) corresponding to GS 45, GS50 and GS58 of the Zadoks scale, respectively. Disease severity corresponds to pycnidia coverage percentages estimated by assessing leaves of 10 to 20 plants per row, and an average of the pycnidia coverage percentages (PC) on the inoculated adult accessions was subsequently used [22]. The first two scoring data were used to calculate the relative area under the disease progress curve (RAUDPC) for quantitative analyses of the temporal differences in disease progress as detailed by Ouaja et al. (2020) [22].

### Phenotypic data analysis

All the phenotypic data were analyzed using the R statistical package [51]. The fourteen phenotypic traits assessed were as follows: TUN16D (severity for TUN06 isolate in 2016), TUN17D (severity for TUN06 isolate in 2017), TUNMeanD (severity for TUN06 isolate averaged across 2016 and 2017), TUN16A (RAUDPC for TUN06 isolate in 2016), TUN17A (RAUDPC for TUN06 isolate in 2017), TUNMeanA (RAUDPC for TUN06 isolate averaged across 2016 and 2017), TM17D (severity for TM220 isolate in 2017), TM17A (RAUDPC for TM220 isolate in 2017), BULK18D (severity for BULK isolates in 2018), BULK18A (RAUDPC for BULK isolates in 2018), DTH16 (days to heading in 2016), DTH17 (days to heading in 2017), DTHmean (days to heading averaged across 2016 and 2017), and PHT (plant height in 2016). The best linear unbiased estimator (BLUE) values for all traits within an individual environment were calculated using the lmer function from the “lme4” package in R and were subjected to subsequent GWAS analysis [52]. Pearson’s correlation coefficients for all the phenotypic traits were assessed using the pairs. panel function in the R package “psych” [53].

### Genotyping and linkage disequilibrium (LD) analysis

A total of 20,279 polymorphic SNPs were generated by Illumina sequencing 373 Tunisian durum wheat accessions using a High-density 90K wheat SNP array (iSelect, San Diego, USA) [37]. After filtering for SNPs with less than 20% of missing data and a minor allele frequency of 5%, 15,944 polymorphic SNP markers were retained. 7,568 SNPs mapped on the SNP-based consensus map of tetraploid wheat [54] were used for GWAS analysis (S1 Table). Linkage disequilibrium (LD) analysis was conducted with 10,264 SNP markers mapped on the RefSeqv1.0 assembly. The 10,264 SNP markers were retained after alignment of the 90K array SNP sequences to IWGSC RefSeq v.1.0 using the following parameters: ‘coverage > 95%, identity > 97%, e-value < 10^−10^. SNPs with multiple mapped locations were removed. To estimate linkage disequilibrium (LD), the square of the correlation coefficient of SNP alleles between loci (r^2^) estimates was calculated using TASSEL software (v.5.2). LD decay plots were generated in R v4.0.3. The half-decay distance was calculated using the estimated maximum value of LD and the Remington model was used to fit a nonlinear model for the relationship between LD decay and distance [55].

### Genome-wide association study and QTL identification

The GWAS was conducted across 373 durum wheat accessions using BLUE estimates of fourteen phenotypic traits (three morphological and eleven disease traits using three isolates) across five environments (TUN06 isolate in 2016 and 2017, as well as their combined, TM220 isolate in 2017, and BULK isolates in 2018) during 2016–2018. Initially, five GWAS models were tested: generalized linear model (GLM), mixed linear model (MLM), and enriched compressed mixed linear model (ECMLM) under the single locus model category and multiple loci mixed model (MLMM), and fixed and random model circulating probability unification (FarmCPU) under the multiple loci model category. Based on the Q-Q plot, the best model (FarmCPU) showing the highest probability of association, was used for association mapping in the Genomic Association and Prediction Integrated Tool (GAPIT) v3.0 package in R [56].

The first three principal components were used to control population structure in the association analysis based on the model fit in the quantile-quantile (Q-Q) plots and the Scree plot [57]. Significant MTAs were identified based on the Bonferroni correction threshold of -log_10_(*P*) > 5.184. The cutoff value for significant MTAs was also confirmed by visualizing the *P*-value distribution in the Q-Q plots. The phenotypic variance explained (PVE) by the significant MTA was estimated by fitting the ordinary least square regression model where phenotype (response variable) was a function of significant marker (independent variable). Significant MTAs were further assessed for their pleiotropic effect and stability, where single locus attributing multiple phenotypic traits was considered in pleiotropy and markers expressed at two or more environments were considered stable. Significant markers were also displayed in the Manhattan plot constructed using the “qqman” package in R [58]. Quantitative trait intervals (QTIs) were defined as a group of MTA upstream and downstream from the most significant MTA (peak position) within an interval window corresponding to whole genome LD decay. A QTL is retained if it contains an MTA for at least one of the TUN06 mean disease trait (TUNMeanA or TUNMeanD). QTL name were assigned using the nomenclature Qstb chromosome—followed by the number of QTL within a chromosome.

### Identifying candidate genes and colocalizing with known QTL

Probe sequence of each significant MTA was aligned to Svevo genome version 1.0 (http://plants.ensembl.org/Triticum_turgidum) for genomic localization. To compare QTL identified in our study with Stb genes/QTL reported in the literature, the corresponding locations of the identified QTL were projected onto Maccaferri et al. (2015) [54] map for cross-reference. QTL within a position over 10 cM from previously reported Stb genes or QTL described in the literature was considered as novel QTL [24, 34, 39]. In addition, putative candidate genes were identified for the four major QTL (PVE>30%) by searching for candidate genes at -/+ 5Mb upstream and downstream to the major QTL peak position on the annotated Svevo genome version 1.0.

Because cloned *Stb* genes encode wall-associated-like-receptor -kinase (WAK) perceiving molecular compounds present in the apoplasm [59–61], candidate genes (CG) search was investigated for functional domains or genes encoding for receptor-like kinases involved in specific gene-for-gene interaction based on three gene ontology (GO) codes; GO:0006468, GO:0016021, and GO:0048046 annotated, respectively, for protein phosphorylation, integral component of membrane, and apoplast domain using BioMart in Ensembl Plants.

## Results

### Phenotypic analysis

**Fig 1:**
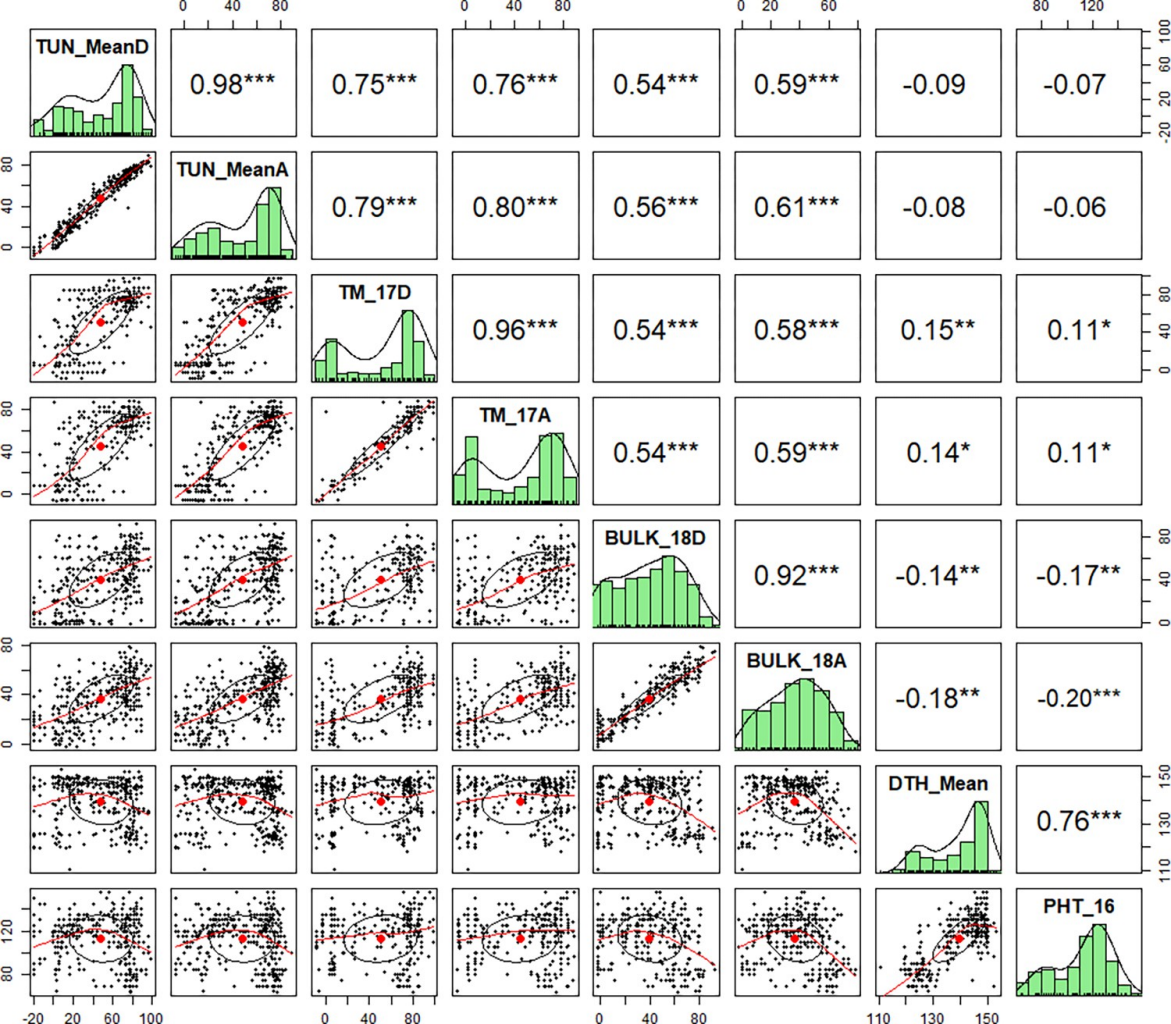
Pearson’s correlation matrix among six Septoria tritici blotch (STB) disease traits and two morphological traits of 373 durum wheat genotypes. Diagonal boxes show the frequency distributions. The above diagonal shows correlation values between traits and their significance while the below diagonal contains the graphical representation of smoothed regression lines across the scatter plot. The correlation matrix was generated using the ‘psych’ package in the R statistical software. TUN, TUN06 isolate; TM, TM220 isolate; BULK, mixture of isolates; DTH, days to heading; PHT, plant height (cm); 16, 2016; 17, 2017; 18; 2018; mean; average of two years of study; D, disease severity (% of pycnidia); A, relative area under disease progress curve (RAUDPC). * Significant at the 0.05 probability level; ** Significant at the 0.01 probability level; ***.

A phenotypic analysis for STB infection on 373 durum wheat panel was carried out with a bulk of five isolates (BULK) during the 2018 cropping season. The percentage disease severity at 37 days post inoculation (D2) and relative area under the disease progress curve (RAUDPC) results using the BULK isolates were compared to the two results obtained in previous experiments using single isolates, TUN06 (in 2016 and 2017 cropping seasons) and TM220 (in 2017 cropping season) (S2 Table). Highly significant correlations (r^2^ = 0.960.98; *p*<0.001) were observed between the disease traits (TM_17D and TM_17A and, TUN_MeanD and TUN_MeanA, respectively) (Fig 1). Interestingly, the inoculation with different isolates, TUN06 and TM220 were significantly and highly correlated (r^2^ = 0.75–0.8; *p*<0.001). STB disease traits measured under TUN06 and TM220 isolate inoculations showed a significant correlation (r^2^ = 0.54–0.61; *p*<0.001) when compared with STB disease traits using the BULK isolates. A discontinuous distribution of the disease traits after inoculation with TUN06 or TM220 was observed revealing a qualitative response to inoculation with a single isolate. However, when the inoculation was carried out with the BULK isolates, the distribution of disease traits showed a quantitative distribution (Fig 1).

STB disease traits in 2016 (r^2^ = -0.12 to -0.21; *p*<0.001–0.05) and in 2018 (r^2^ = -0.14 to -0.19; *p*<0.001–0.01) were significantly and negatively correlated with the days to heading (DTH). Similarly, STB disease traits in 2018 were significantly and negatively correlated with DTH and plant height (PHT) (r^2^ = -0.17 to -0.20; *p*<0.001–0.01) (S1 Fig). Genotypes having a shorter flowering time and smaller plant height have a higher STB infection, i.e., late flowering and tall plants escaped the disease and therefore appeared more resistant. It was previously assumed that plants with shorter flowering time and plant height would favor the occurrence and progression of the disease [62]. However, STB disease traits in 2017 were significantly and positively correlated with DTH (r^2^ = 0.14 to 0.16; *p*<0.01–0.05) and PH (r^2^ = 0.11, p<0.05). Favorable humid conditions in 2017 could have swapped the commonly expected negative correlation between STB and DTH.

### Marker trait associations (MTAs)

**Fig 2:**
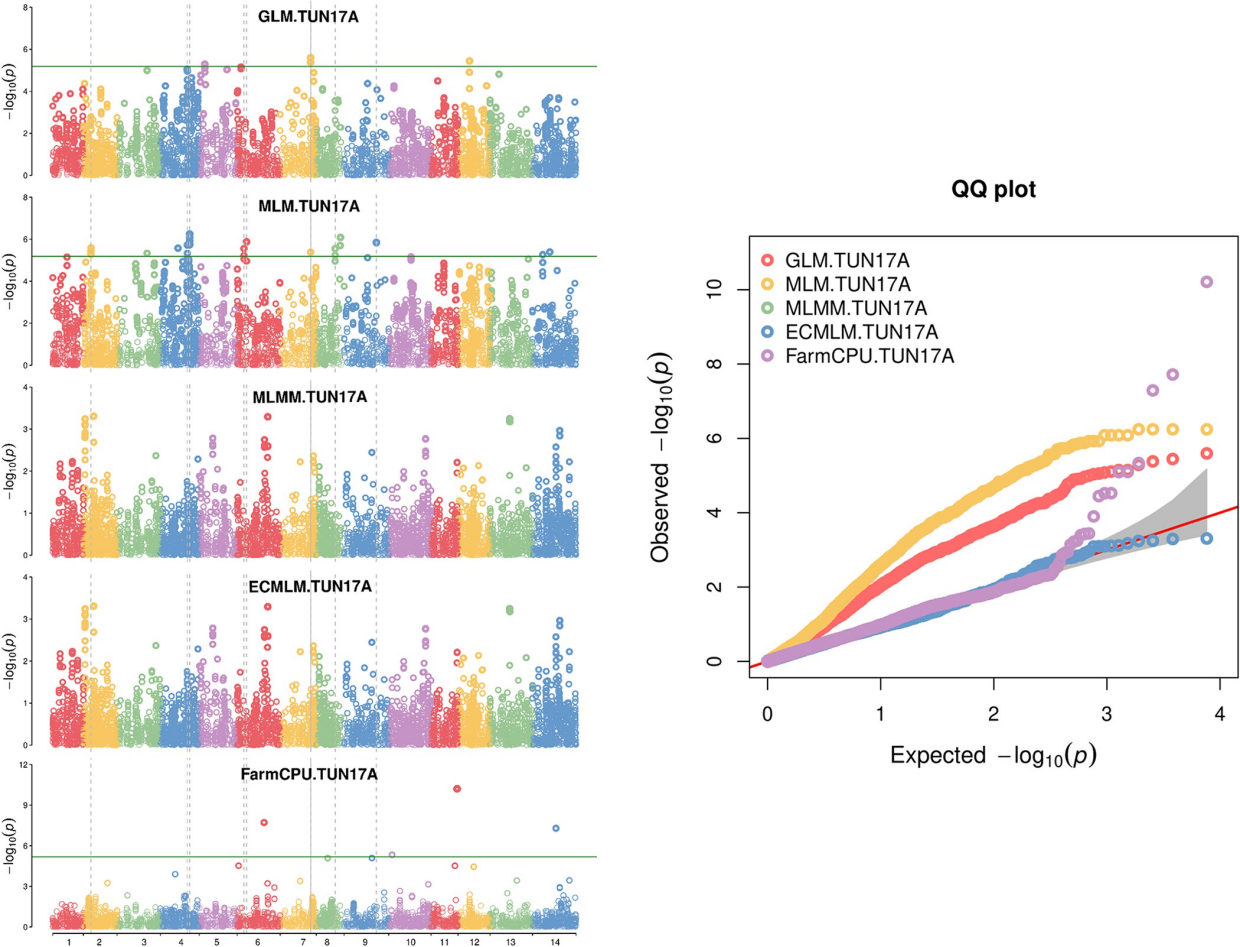
Multiple Manhattan plots (left) and quantile-quantile (Q-Q) plot (right) comparing five different GWAS models, for TUN17A (relative area under disease progress curve causing Septoria tritici blotch (STB) during 2017). The green horizontal line in the Manhattan plot correspond to Bonferroni correction of -log_10_(*p*) >5.18. GLM, generalized linear model; MLM, mixed linear model; MLMM, multiple loci mixed model; ECMLM, enhanced compressed mixed linear model; FarmCPU, fixed and random model circulating probability unification. FarmCPU was the model retained in this study.

**Fig 3:**
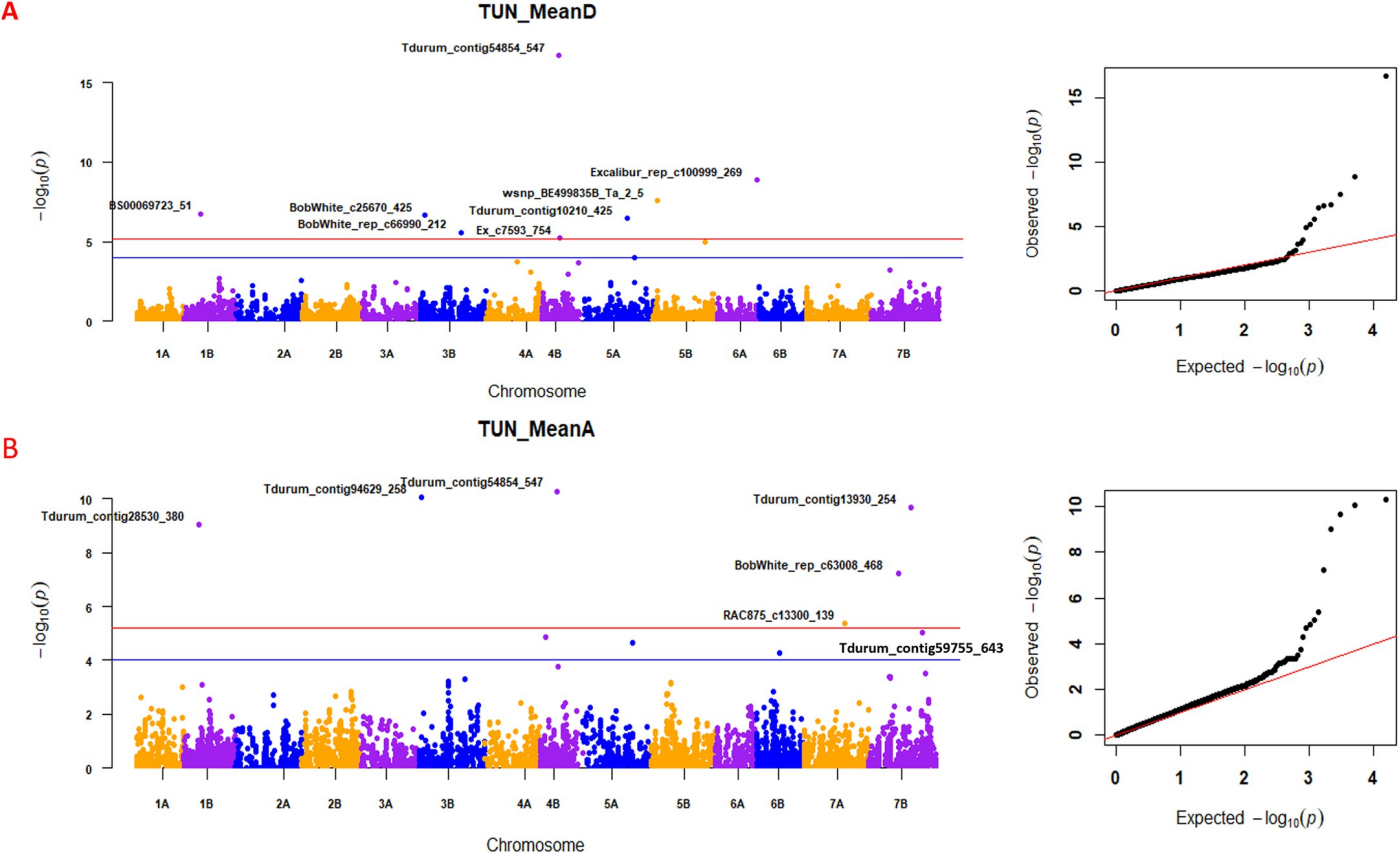
Manhattan plot (left) and quantile-quantile (Q-Q) plot (right) for mean Septoria tritici blotch (STB) disease scores after inoculation with TUN06 isolate during 2016 and 2017. (A) Mean pycnidial coverage at day 35–37 dpi (TUNMeanD) and (B) mean rAUDPC values (TUNMeanA). A The blue and red horizontal lines in the Manhattan plot correspond to the -log_10_(*P*) value of 4.0 and 5.18 (Bonferroni correction), respectively. Quantitative trait loci (QTLs) associated with significant single nucleotide polymorphism (SNPs) above Bonferroni correction threshold line are displayed in the red font in the box.

The GWAS was performed with 7,638 SNP markers after removing SNP markers with unknown chromosome allocation loci (S2 Table). Disease severity (D) and RAUDPC measured for the three different inoculums (TUN06, TM220, and BULK) were used to conduct the GWAS. DTH and PHT were also added to the GWAS. Among different GWAS models, FarmCPU model showed reliable results and presented low spurious associations compared to the other models GLM, MLM, MLMM, and ECMLM (Fig 2) [56]. This model has identified significant MTA with a high probability surpassing the Bonferroni threshold of -log_10_(*p*) = 5.18 (S3 Table). Individual marker *p*-values and FDR adjusted *p* values for GWAS scan on STB are reported in S3 Table. Manhattan plots and QQ plots for the associations between markers and STB mean responses to TUN06 were described in Fig 3. Fifty-one significant MTA were identified for STB resistance on 11 different chromosomes (S3 Table). Thirty-three, fifteen and three MTA were identified for the single isolates TUN06, TM220, and the BULK, respectively. For the isolate TUN06 inoculated during two cropping seasons, the number of MTA were higher in 2017 (12) than in 2016 (7). Five and three MTA were identified for DTH and PHT each on three chromosomes, respectively (S3 Table) and the respective Manhattan plots and QQ plots are presented in S2 and S3 Figs.

### Linkage disequilibrium and QTL identification

**Fig 4:**
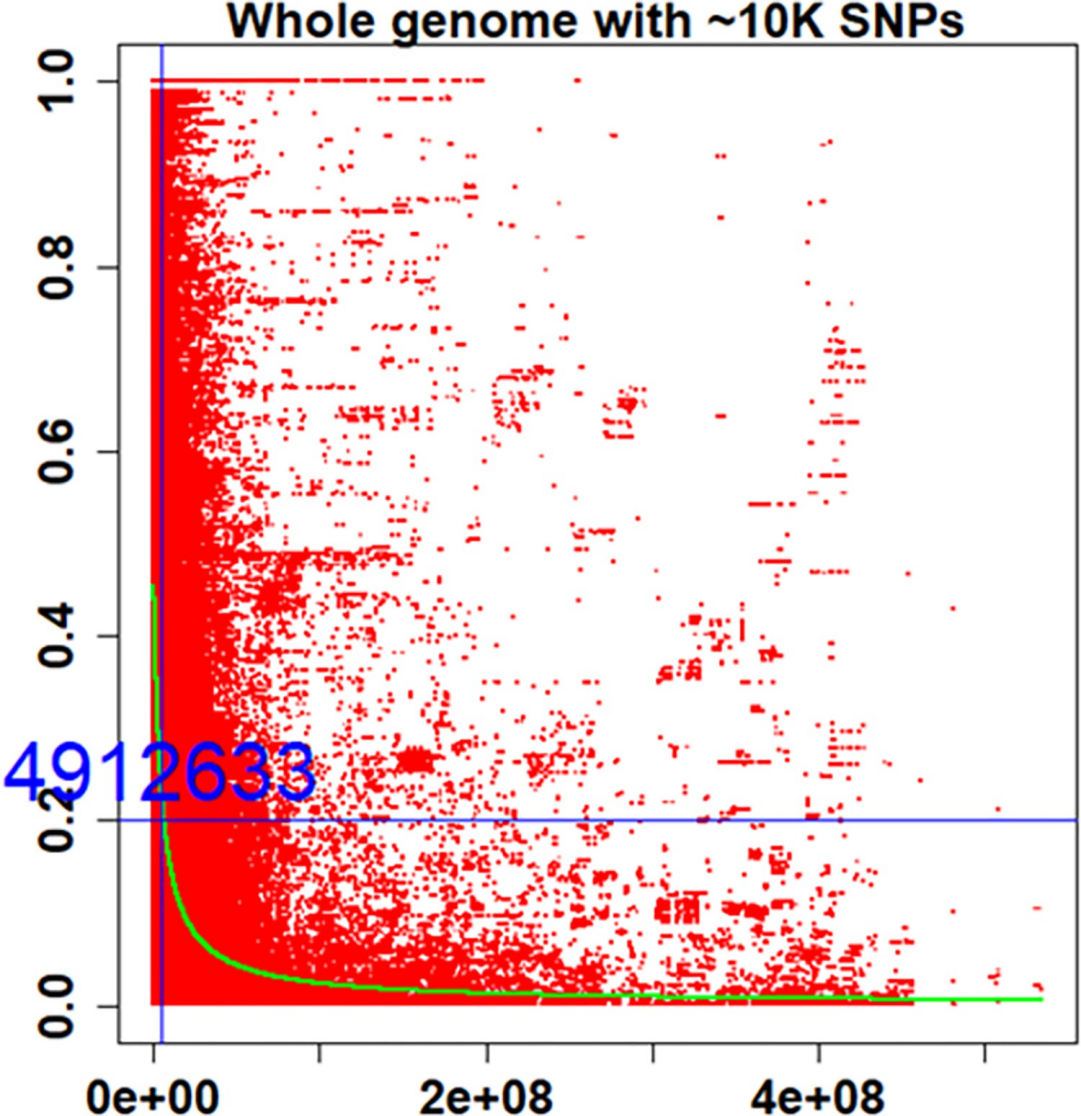
Intra-chromosomal linkage disequilibrium (LD) decay plot as a function of genetic distance (cM). LD decay assessed in a Tunisian old durum wheat panel of 367 accessions and 6 modern cultivars and 10.264SNP markers. LD estimates MTA are reported as squared correlations of allele frequencies (r2). Inter-marker genetic distances are from mapping position on RefSeqv1.0 assembly.

LD decay was investigated using 10,264 SNP markers across the whole genome of IWGSC RefSeqv1.0 through fitted regression and box plot distribution. The half LD decay value (r^2^ = 0.2) was noted at 4.9 Mb (Fig 4).

For assigning MTA into a QTL, MTA located on the same chromosome were grouped at +/- 5 Mb interval (whole genome LD decay) from significant MTA with the highest PVE values (Table 1). Among 51 significant disease trait MTA, 30 assigned to 7 chromosomes were retained to build a total of 11 QTL with optimal PVE values varying from 4.6 to 58.2%. Four QTL consisted of a single MTA and seven QTL consisted of at least two MTA. A QTL that contributed less than 30% of the phenotypic variance (or quantitative effect) for any associated phenotypic trait was considered a minor effect, while those contributing greater than 30% were considered major effect QTLs. Four QTL for STB resistance, *Qstb_1B*.*1*, *Qstb_4B*.*1*, *Qstb_5A*.*1*, and *Qstb_7B*.*2*, had major effects accounting for 33, 37.7, 39.7 and 58.2% of the PVE, respectively (Table 1). The rest were minor QTL with PVE varying from 4.6 to 26.7%. Among 11 QTL, four QTL (*Qstb_3B*.*3*, *Qstb_4B*.*1*, *Qstb_5A*.*1*, and *Qstb_7B*.*1)* were stable (expressed in two or more environments) and five QTL (*Qstb_3B*.*3*, *Qstb_4B*.*1*, *Qstb_5B*.*1*, *Qstb_7B*.*1*, and *Qstb_7B*.*2)* displayed pleiotropic effect controlling two or more phenotypic traits. *Qstb_1B*.*1* was stable and pleiotropic. From 11 QTL conferring STB resistance, two QTL, *Qstb_3B*.*3* and *Qstb 7B*.*2*, controlled resistance to both inoculum TUN06 and TM220. However, no QTL controlling resistance to both TUN06 and BULK inoculum was detected. *Qstb_7B*.*2* also controlled heading date. However, none of the identified QTL for resistance was associated with plant height. *Qstb_7B*.*1* and *Qstb_7B*.*2* were proximal on chromosome 7BS and at a distance varying from 20 to 37 cM.

**Table 1:** Summary of identified quantitative trait loci (QTLs) associated with Septoria tritici blotch (STB) resistance in durum wheat across different isolates of *Zymoseptoria tritici* and morphological traits and their respective candidate gene annotation (*).

QTL	Trait	SNP	Chromosome	Position (Mb)	Position (cM)	PVE (%)	Known QTL and Stb genes	Gene ID (distance in Mb)	Description (extracellular domain)
*Qstb_1B*.*1*	TUN16D	Tdurum_contig28530_380	1B	423014475	50.2001	4.4			
	TUNMeanA	Tdurum_contig28530_380	1B	423014475	50.2001	9.6			
	TM17D	Tdurum_contig28530_380	1B	423014475	50.2001	7.8			
	TUN16A	IAAV9039	1B	474657310	57.5999	**33.0**		TRITD1Bv1G152960 (0,67)	Receptor-like protein kinase SRF-like (SPARK domain)
*Qstb_1B*.*2*	TUNMeanD	BS00069723_51	1B	692274202	53.2000	**4.6**	*QStb*.*teagasc-1B*.*2* [34]		
*Qstb_3B*.*1*	TUNMeanD	BobWhite_c25670_425	3B	19109100	15.4001	**11.1**	** **		
*Qstb_3B*.*2*	TUNMeanA	Tdurum_contig94629_258	3B	8997703	7.3999	**13.3**	** **		
	TUNMeanD	BobWhite_rep_c66990_212	3B	717253844	131.4000	0.2	*QstbIsa_af3B* and *QTL4* [24]		
*Qstb_3B*.*3*	TUN17A	BobWhite_rep_c66990_212	3B	717253844	131.4000	-0.1	*QStb*.*teagasc-3B*.*3* [34]		
	TUN17D	Kukri_c14967_836	3B	759477151	149.6000	8.2			
	TM17A	Kukri_c14967_836	3B	759477151	149.6000	**16.3**			
	TM17A	Tdurum_contig9738_262	3B	807121554	188.2000	8.5			
*Qstb_4B*.*1*	TUN16D	Tdurum_contig54854_547	4B	425728896	55.6000	32.8	*Qstb*.*Isa_fb-4B* [24]		
	TUNMeanD	Tdurum_contig54854_547	4B	425728896	55.6000	31.4	*QStb*.*teagasc-4B*.1 [34]		
	TUN16A	Tdurum_contig54854_547	4B	425728896	55.6000	34.1	*MQTL18* [39]	not detected	
* *	TUNMeanA	Tdurum_contig54854_547	4B	425728896	55.6000	**37.0**	** **		
*Qstb_5A*.*1*	TUNMeanD	Tdurum_contig10210_425	5A	531759920	135.2000	0.7	*QTL*.*9* [39] *QStb*.*teagasc-5A*.1 [34] *qSTB*.*5* [40]		
	TUN16D	IAAV8042	5A	577669182	158.9001	**39.7**		TRITD5Av1G217560 (0,66)	Receptor-like protein kinase (sugar-binding protein domain)
*Qstb_5B*.*1*	TUNMeanD	wsnp_BE499835B_Ta_2_5	5B	20749661	16.6999	**8.1**	*QStb*.*teagasc-5B*.*1* [34]		
* *	TUN17A	wsnp_BE499835B_Ta_2_5	5B	20749661	16.6999	8.0			
*Qstb_6A*.*1*	TUNMeanD	Excalibur_rep_c100999_269	6A	608156746	126.6999	13.6			
*Qstb_7B*.*1*	TUN17A	RAC875_c1742_2710	7B	583835818	114.2000	**26.7**	*QStb*.*teagasc-7B*.*1* [34]		
	TUN17D	RAC875_c1742_2710	7B	583835818	114.2000	20.1	*QStb*.*ipk-7B* [24]		
	TUNMeanA	Tdurum_contig13930_254	7B	628088316	132.8001	7.3			
* *	TM17D	Tdurum_contig59755_643	7B	686789226	169.8000	3.4	*Stb8* [63]		
	TUNMeanA^e^	Tdurum_contig59755_643	7B	686789226	169.8000	4.2	*QStb*.*teagasc‐7B*.*1* [34]		
	TM17A	Tdurum_contig59755_643	7B	686789226	169.8000	2.8	*Qstb*.*renan-7B* [64]		
*Qstb_7B*.*2*	TM17A	BS00049961_51	7B	692274202	179.6000	**58.2**		TRITD7Bv1G222460 (2,25); TRITD7Bv1G224050 (1,1)	Receptor-like protein kinase (DUF3475); Leucine-rich repeat domain superfamily (matrix-binding glycoproteins)
	TM17D	CAP12_c1587_142	7B	692454773	179.5999	57.0			
* *	DTHMean	Kukri_rep_c70199_800	7B	703142411	176.7000	34.1			

^a^TUN, TUN06 isolate; TM, TM220 isolate; DTH, days to heading; PHT, plant height (cm); 16; 2016; 17, 2017; mean; average of two years of study; D, disease severity; A, relative area under disease progress curve (RAUDPC)

^d^ Percentage of phenotypic variance (PVE) explained by the marker

^e^MTA with -Log_10_P value (5.01) less than Bonferroni threshold

^f^ Gene annotations were carried out on MTA with effect >30% using the reference *Triticum turgidum* Svevo genome

^g^PVE in bold correspond to the peak position of the QTL

### QTL colocalizing with known QTL and *Stb* genes

Among 11 QTL identified in this study, 7 QTL colocalized with known Stb/QTL for resistance (Table 1). The minor QTL, *Qstb_1B*.*2* (PVE: 4.6%), on chromosome 1BL at 692 Mb was 30 Mb away from *QStb*.*teagasc‐1B*.*2* identified in bread wheat in the interval of 648.45–662.72 Mb [34]. *Qstb*_*3B*.*3* in the interval of 717 Mb (131 cM) - 807 Mb (188 cM) on chromosome 3BL colocalized with *QstbIsa_af3B* and, *QTL4*, identified in previous studies [24, 39]. The major QTL *Qstb*_*4B*.*1* (PVE: 37%) at the peak position of 425 Mb (55.6 cM) on chromosome 4BS overlapped with *Qstb*.*Isa_fb-4B* and *QStb*.*teagasc-4B*.1 and was adjacent to *MQTL16* [24, 34, 39].

The major *Qstb*_*5A*.*1* at the interval position of 521–577 Mb (135–158.9 cM) on chromosome 5AL overlapped with *QTL*.*9* laying between 159.3 and 159.8 cM [39], *QStb*.*teagasc-5A*.1 at the interval of 0–138 Mb [34] and the GWAS identified QTL *qSTB*.*5* in durum wheat at 135 cM [40]. *Qstb_5B*.*1* identified on chromosome 5B at the peak position of 20.7 Mb (16.7 cM), localized within the large QTL, *QStb*.*teagasc-5B*.*1* laying between 17.97 Mb and 690 Mb [34]. *Qstb*_*7B*.*1* (PVE: 26.7%) mapped at 583–628 Mb (114–132 cM) interval on chromosome 7BL overlapped with two large QTL, *QStb*.*teagasc-7B*.*1* laying from 106 to 625 Mb and *QStb*.*ipk-7B* laying between 79 and 143 cM [24]. The major QTL, *Qstb_7B*.*2* accounting over 58% for PVE at the interval between 686 Mb (170 cM) and 703 Mb (177 cM) also overlapped gene/QTL accounting for major resistance including *Stb8* [63], *QStb*.*teagasc‐7B*.*1* [34], and *Qstb*.*renan-7B* [64] on 7BL chromosome. In fact, the later QTL lay between 160 and 170 cM accounted for a maximum of 38% of PVE. Similarly, *QStb*.*teagasc-7B*.1 identified by Riaz et al. (2020) [34] had the highest contribution to the PVE (29%) among 25 identified QTL.

### Identification of candidate genes

Cloned *Stb* genes were identified to encode for cell wall-associated receptor kinase (WAK) with extracellular domain involved at the initial stages of pathogen recognition during *Z*. *tritici* infection [59–61]. With the hypothesis that major QTL may encode for *Stb* genes, gene identification was investigated by searching within an interval of 5 Mb (corresponding to KP decay) upstream and downstream from the peak position of the four major QTL identified (PVE>30%) for receptor like kinase (RLK) and receptor like protein (RLP) carrying an extracellular domain (Table 1**)**. Receptor-like kinase candidate genes were attributed to three (*Qstb_1B*.*1*, *Qstb_5A*.*1*, and *Qstb_7B*.*2*) of the four identified major QTL. Interestingly, a candidate gene encoded for a leucine-rich repeat (LRR-RLK) with a sugar binding domain was detected at 0.66 Mb and 1.1 Mb from the peak position of *Qstb_5A*.*1* and *Qstb_7B*.*2*, respectively.

## Discussion

We previously demonstrated a high genetic diversity of Tunisian durum wheat germplasm using 10 SSR and 286 SNPs and analyzed its response to STB infection during two consecutive years 2016 and 2017 under field inoculation with two different isolates TUN06 and TM220 [8, 50]. Considering that Tunisian germplasm harbors valuable sources of resistance, it was important to exploit a high-density SNP array for GWAS on STB resistance to identify new putative QTL.

This current study based on a high throughput genotyping array and a high-density SNP map implemented in durum wheat provided us with an excellent approach to analyze a larger pool of durum wheat accessions to uncover novel QTL for resistance to STB. This association mapping was conducted on a highly diverse durum wheat panel [8], and limited population structure [50], thus making it a good source for association mapping for resistance to STB. In this study, an additional inoculation with a bulk of five isolates was also investigated for GWAS, as well as the single inoculation with isolates TUN06 and TM220. A quantitative distribution of disease response to *Z*. *tritici* was observed for the BULK inoculum compared to qualitative distributions revealed with single isolates TUN06 and TM220. This quantitative response could be explained by the interaction and compensatory effect between different resistance genes responding to different *Z*. *tritici* isolates in the mixture thereby reducing the effect of the resistance genes. In fact, a limited number of MTA (3) was obtained after inoculation with the bulk of isolates compared to single isolate (>6). Infection with a single isolate is well untangled in open field infestation for QTL analysis and this study also supports the importance of inoculation with monosporal culture in the field to study the genetics for resistance to STB [28, 49]. It has been demonstrated that in *Z*. *tritici*-wheat pathosystem, both qualitative and quantitative resistances follow gene-for-gene interaction [65]. The variation from qualitative to quantitative response also depends on sequence variability within the avirulence gene (Sanchez-Vallet personal communication). Therefore, the effect of the major QTL observed in our study could be also a consequence of the non-adaptive isoform of the avirulence gene of the Tunisian TUN06 isolate.

In the present study, LD analysis conducted with 10,264 SNPs probes selected for a single localization and high probability alignment in the IWGSC RefSeq v.1.0 genome provided an accurate picture of LD decay in the average interval of 5 Mb. The results indicated that confidence mapping interval is at least two times less in magnitude as compared to those obtained in previous studies with durum wheat landraces. In previous studies, linkage disequilibrium was found to decay within 2.2 cM (at r^2^ = 0.3) in a set of 183 tetraploid accessions from the Mediterranean region [46] and within 1.5–6.0 cM (at r^2^ = 0.2) in a panel of 318 Ethiopian durum wheat genotypes [40]. The LD decay estimated with 500 polymorphic SNPs in a small set of Tunisian durum wheat panel composed of 28 durum wheat landraces and 15 modern cultivars corresponded to 3.5 Mb (at r^2^ = 0.2) [9]. Recently, a study on 235 Tunisian durum wheat accessions indicated an average LD half decay was equal to 4.6 and 5.4 Mb for genome A and genome B, respectively [10]. This weak LD relies, in particular, on the high level of admixed genotypes in Tunisian durum wheat landraces panel (22 and 18.72%) observed in Tunisian durum landraces [10, 50] and thereby reducing the confidence interval to 50 times less (0.01 cM) than it was reported for landrace populations [66].

Taking into consideration of combined TUN06 disease scoring measured in 2016 and 2017, we identified 11 QTL for resistance to STB on 7 chromosomes (Table 1). Four major QTL were detected with PVE varying from 33 to 58.2% and seven minor QTL with PVE varying from 4.6 to 26.7%. As previously described, the resistance to this pathogen follows a qualitative and quantitative nature [40, 41, 44, 49, 67]. Previous GWAS conducted either on seedling or adult stage upon single isolate inoculation detected only minor QTL with PVE not exceeding 29% [34, 38, 39, 41–43]. Here, the identification of major QTL reaching up to 58% probably relied on the highly virulent isolate used. Previously, the virulent single isolate TUN06 also allowed the identification of a major QTL (PVE: 61.6%) co-localized with *Stb9* in an old durum wheat landrace Agili39 [49].

Two of the identified QTL encoded for both resistance to TUN06 and TM220 isolates and could explain the high and significant correlations of these isolates for the disease traits. The broad spectrum resistance identified here could be due to the non-adaptation of the Tunisian *Z*. *tritici* populations to the small-scale cultivation of old landraces practiced by traditional farmers [50]. Nevertheless, in this study, >100 old landraces carried at least three QTL for STB resistance (S4 Table) supporting their broad resistance spectrum, as it was also identified in Agili39 [49].

More significant MTA were observed in 2017 with the same isolate TUN06 than in 2016. In 2017, higher STB severity was shown compared to 2016 due to more conducive weather conditions to STB infection at the regional Research center on major crops (CRRGC) Beja [22], leading to better discrimination between susceptible and resistant genotypes. Therefore, the STB field assay during favorable years with virulent isolates at CRRGC Beja could facilitate marker identification for STB resistance in durum wheat and their use for gene pyramiding [22, 23, 49].

The assessment of whether the identified STB QTL are at the same position as previously detected ones is always difficult due to the use of different types of markers, mapping populations and species (bread versus durum wheat). In this study, co-localization of our STB QTL with previous *Stb* genes and QTL encoding for STB resistance was assessed by comparing the localization of Stb/QTL flanking SSR markers with mapped SSR/SNP markers in tetraploid consensus map [54]. With the assumption that *Stb* genes identified in bread wheat are functional in durum wheat i.e. *QTl*.*2B*.*2* identified in durum wheat Agili39 co-localized with *Stb9* [49], it became comfortable to compare QTL with the mapped chromosomal location of previously known genes/QTL mapped on bread wheat consensus map. In this study, 7 of the 11 identified QTL overlapped or were adjacent to regions with known QTL that have previously been identified [24, 34, 39]. Out of the 11 QTL identified in our study, only one QTL corresponded to known Stb resistance gene, *Stb8* identified in bread wheat [63]). The major QTL *Qstb 5A*.*1* and *Qstb 5B*.1 identified in this study colocalized with minor QTL identified in European bread wheat. McDonald and Mundt (2016) [47] suggested that due to the large scale bread wheat cultivation of several resistant varieties in Europe, *Stb* genes were less effective against *Z*. *tritici* populations. *Qstb 5A*.*1* was the only QTL colocalizing with *qSTB*.*5*, one of the five QTL identified by Kidane et al. (2017) [40] using GWAS on Ethiopian durum wheat landraces. This result highlighted the diversity of Tunisian durum wheat landraces in discovering QTL associated with resistance to Tunisian *Z*. *tritici* isolates.

In this study, four QTL were considered as novel, *Qstb_1B*.*1*, *Qstb_3B*.*1*, *Qstb_3B*.*2*, and *Qstb_6A*.*1*. The major QTL *Qstb_1B*.*1* (PVE^2^ = 33%) mapped at 423–474 Mb (50–57 cM) was more than 10 cM apart from *MQTL*.*2* mapped at 36–40 cM [39]. *Qstb_3B*.*1* and *Qstb_3B*.*2*, controlling minor resistance (PVE^2^< 16%) were located at the distal region of chromosome 3BS (8.9 Mb; 0.7 cM and 19 Mb; 15.4 cM, respectively), were at least 20 cM apart from *Qstb-3B*.*1* [34] and did not overlap with QTL described in the literature. The *Qstb_6A*.*1* at the peak position of 608 Mb (126.7 cM) is at 300 Mb from *QStb*.*teagasc-6A*.*1* [34] and 80 cM from *Stb15*.

The release of the reference durum wheat cv. Svevo genome (http://plants.ensembl.org/Triticum_turgidum) and its functional annotation in the Ensembl Plants made possible for the identification of putative candidate genes across significant QTL for resistance to STB disease. Unlike several disease-resistance genes encoding intracellular nucleotide-binding leucine-rich-repeat proteins (NLRs), cloned *Stb6*, *Stb16q* and *Stb15* genes were found to encode a different class of genes, i.e. a wall-associated-like-receptor-kinase (WAK) perceiving molecular compounds present in the apoplasm [59–61]. In this study, candidate gene identification was investigated for four major QTL (PVE>30%) with the assumption that they encode for putative receptor-like-kinase (RLK) or receptor like protein (RLP) controlling gene-for-gene interaction to *Z*. *tritici* in Svevo genome. Receptor-like kinases (RLK) and receptor-like proteins (RLP) located on the cell surface are categorized into various sub-families, such as leucine-rich repeat RLK (LRR-RLK) and wall-associated kinases (WAK) encoding **f**or major resistance genes [68]. RLK candidate genes were attributed to three major QTL (*Qstb_1B*.*1*, *Qstb_5A*.*1*, and *Qstb_7B*.*2*). Two candidate genes encoding for receptor like kinase; *TRITD7Bv1G224050* and *TRITD7Bv1G222460* were identified at +/-5 Mb interval from the most significant MTA of *Qstb_7B*.*2* (Table 1). The WAK gene *TRITD7Bv1G222460* contained a serine/threonine kinase and an extracellular DUF domain, as observed for the major gene *Stb16q* [60]. However, at a closer distance (1.1 Mb), *TRITD7Bv1G224050* encoded for an LRR-RLK which belongs to a subfamily involved in the response against apoplastic leaf pathogen. In addition, *TRITD5Av1G217560* at 0.66 Mb from the peak position of *Qstb 5A*.*1* and *TRITD7Bv1G224050* at 1.1 Mb from the peak position of *Qstb7B*.*2* shared similarities with Stb proteins i.e. they corresponded to transmembrane proteins with extracellular domains with sugar binding function and intracellular kinase. It was suggested that cloned Stb genes possess an extracellular domain bind mannose, a building block of mannan found in cell walls of fungi [61].

## Conclusion

In this study, GWAS using a collection of diverse durum wheat landraces enabled the identification of four major QTL including a novel QTL, *Qstb1B*.*2* and three minor QTL were novel. SNP markers associated with these major QTL could have a powerful impact on genomic prediction and marker-assisted selection in durum wheat breeding programs. Candidate genes involved in pathogen recognition were identified. The function of these genes in STB resistance remain to be investigated. Their characterization will enhance molecular understanding of the wheat-*Z*.*tritici* interaction. A functional approach could include the development and characterization of CRISPR/Cas9-based knockouts from the resistant material [69]. The genetic material could also be exploited for gene cloning especially for *Qstb_7B*.*2*, a major QTL explaining up to 58.2% of the phenotypic variation for STB resistance on chromosome 7BL.

## Supporting information

S1 Table Genotypic data for 373 durum wheat genotypes in the association mapping panel. (XLSX)

S2 Table The raw phenotypic data used in this GWAS analysis. (XLSX)

S3 Table List of 59 significant marker-trait associations (MTAs) associated with Septoria tritici blotch (STB) resistance in durum wheat across different isolates of *Zymoseptoria tritici* and morphological traits. (XLSX)

S4 Table Major QTL allele and resistance color shade of 372 durum wheat genotypes. (XLSX)

S1 Fig Pearson’s correlation matrix among ten Septoria tritici blotch (STB) disease traits and four morphological traits of 373 durum wheat genotypes. Diagonal boxes show the frequency distributions. The above diagonal shows correlation values between traits and their significance while the below diagonal contains the graphical representation of smoothed regression lines across the scatter plot. The correlation matrix was generated using the ‘psych’ package in the R statistical software. TUN, TUN06 isolate; TM, TM220 isolate; BULK, mixture of isolates; DTH, days to heading; PHT, plant height (cm); 16, 2016; 17, 2017; 18; 2018; mean; average of two years of study; D, disease severity (% of pycnidia) A, relative area under disease progress curve (RAUDPC). * Significant at the 0.05 probability level; ** Significant at the 0.01 probability level; *** Significant at the 0.001 probability level.
(PPTX)

S2 Fig Manhattan plot (left) and quantile-quantile (Q-Q) plot (right) for days to heading (DTH) in 2016 (DTH_16; A), 2017 (DTH_17; B), and mean of both of both years (DTH_mean; C). The blue and red horizontal lines in the Manhattan plot correspond to the -log_10_(*P*) value of 4.0 and 5.18 (Bonferroni correction), respectively.
(PPTX)

S3 Fig Manhattan plot (left) and quantile-quantile (Q-Q) plot (right) for plant height (PHT) in 2016. The blue and red horizontal lines in the Manhattan plot correspond to the -log_10_(*P*) value of 4.0 and 5.18 (Bonferroni correction), respectively.
(PPTX)

S4 Fig Structure and kinship analyses in the diversity panel. Samples’ relationship in the three-dimensional space of the first three principal components derived from a principal component analysis (PCA) (A); scree plot retaining three principal components as determined by locating the point at which the graph shows a distinct change in the slope (B); the distribution of estimated kinship values follows a normal distribution (turquoise curve) (top left) and the pairwise kinship values depicted in increasing tones of red with the clustering tree outside the matrix (C).
(PPTX)
