# Supplementary figures and images for: Genome-wide association study reveals major loci for resistance to septoria tritici blotch in a Tunisian durum wheat collection
Source: PLoS One. 2025 Feb 6;20(2):e0310390. doi: 10.1371/journal.pone.0310390 (PMC11801541; doi:10.1371/journal.pone.0310390)

## Slide 1
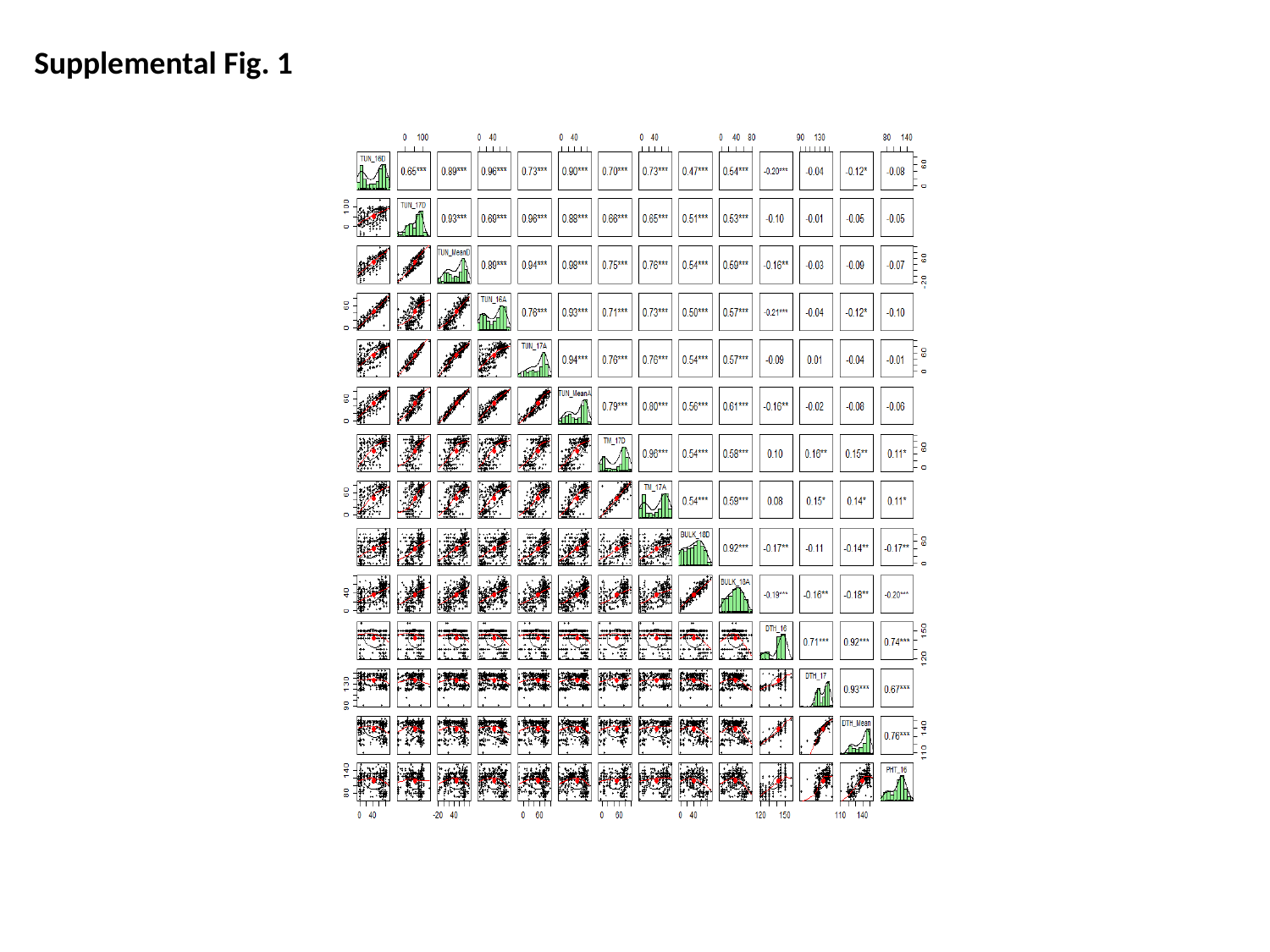

Supplemental Fig. 1

Supplement: S1 Fig — Diagonal boxes show the frequency distributions. The above diagonal shows correlation values between traits and their significance while the below diagonal contains the graphical representation of smoothed regression lines across the scatter plot. The correlation matrix was generated using the ‘psych’ package in the R statistical software. TUN, TUN06 isolate; TM, TM220 isolate; BULK, mixture of isolates; DTH, days to heading; PHT, plant height (cm); 16, 2016; 17, 2017; 18; 2018; mean; average of two years of study; D, disease severity (% of pycnidia) A, relative area under disease progress curve (RAUDPC). * Significant at the 0.05 probability level; ** Significant at the 0.01 probability level; *** Significant at the 0.001 probability level. (PPTX) [file pone.0310390.s005.pptx]

## Slide 1
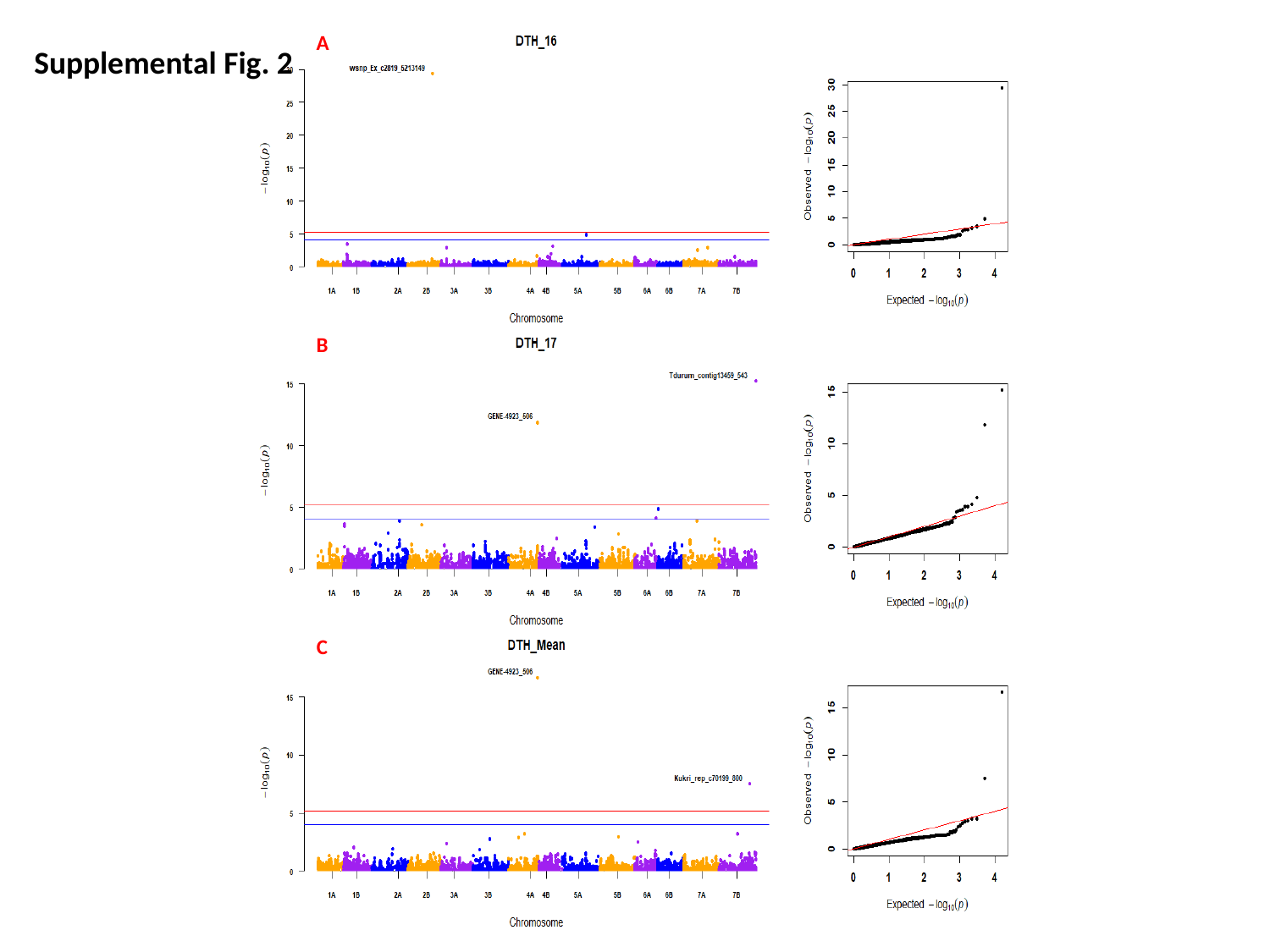

A
B
C
Supplemental Fig. 2

Supplement: S2 Fig — Manhattan plot (left) and quantile-quantile (Q-Q) plot (right) for days to heading (DTH) in 2016 (DTH_16; A), 2017 (DTH_17; B), and mean of both of both years (DTH_mean; C). The blue and red horizontal lines in the Manhattan plot correspond to the -log10(P) value of 4.0 and 5.18 (Bonferroni correction), respectively. (PPTX) [file pone.0310390.s006.pptx]

## Slide 1
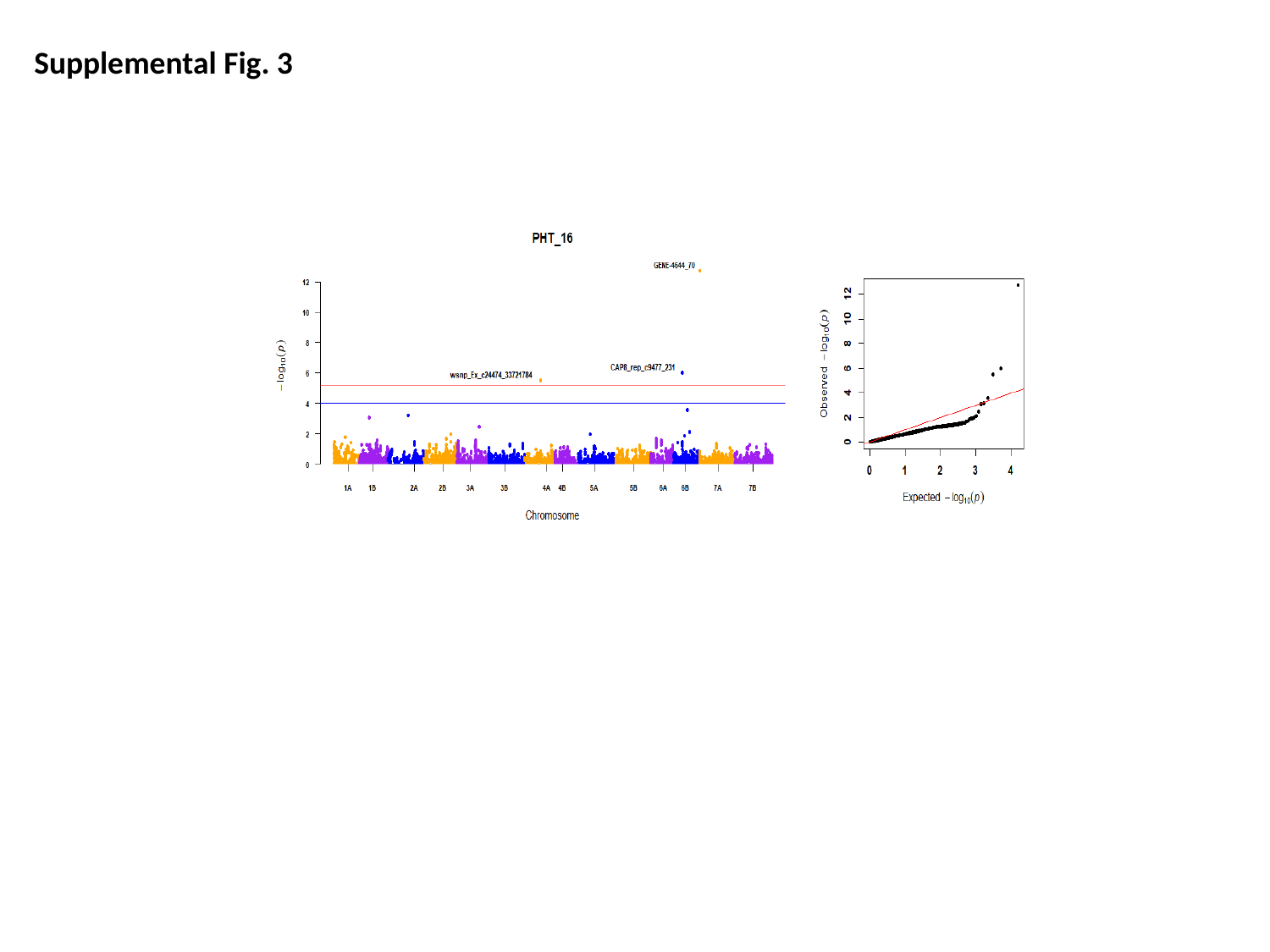

Supplemental Fig. 3

Supplement: S3 Fig — Manhattan plot (left) and quantile-quantile (Q-Q) plot (right) for plant height (PHT) in 2016. The blue and red horizontal lines in the Manhattan plot correspond to the -log10(P) value of 4.0 and 5.18 (Bonferroni correction), respectively. (PPTX) [file pone.0310390.s007.pptx]

## Slide 1
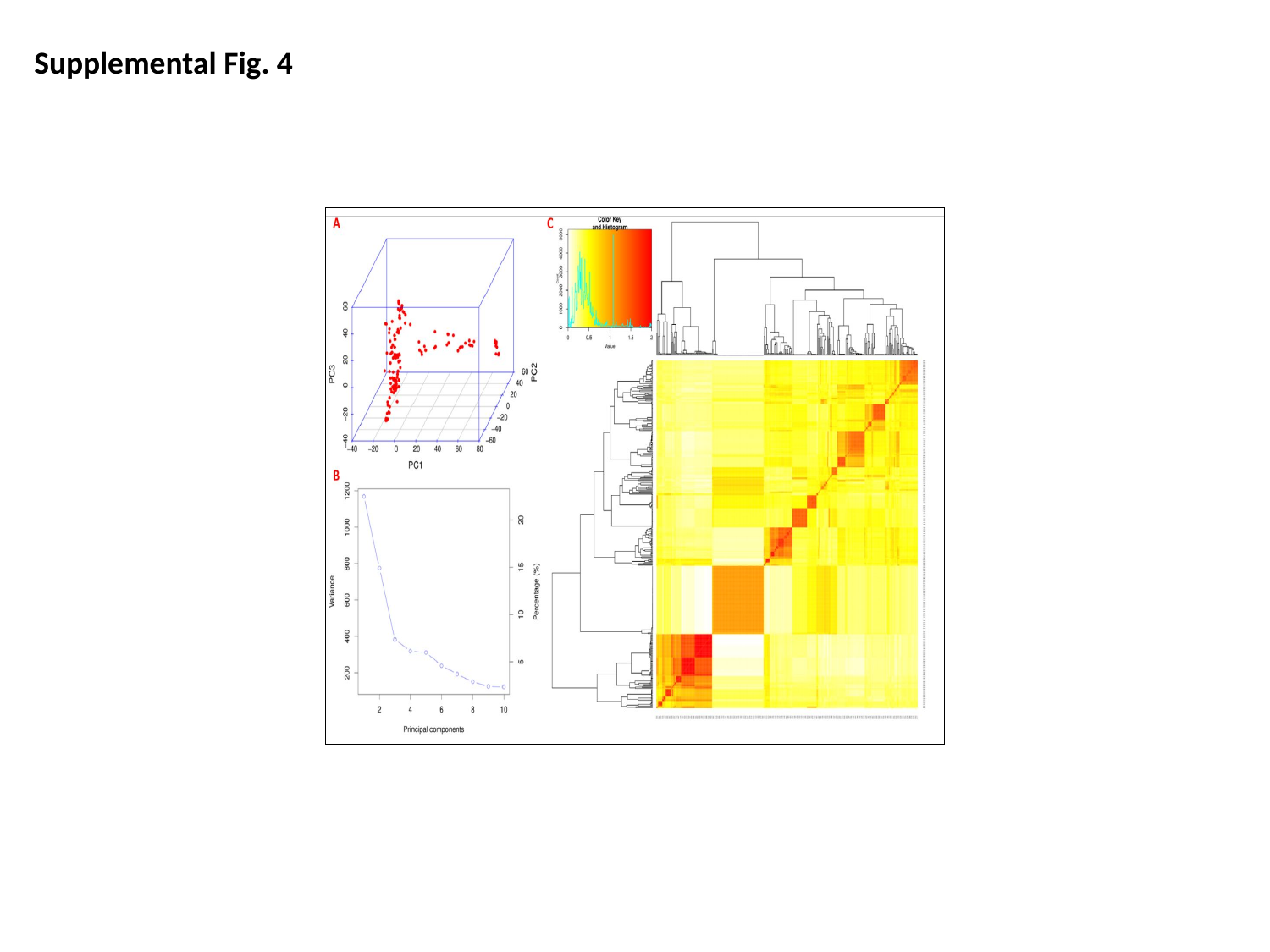

Supplemental Fig. 4

Supplement: S4 Fig — Samples’ relationship in the three-dimensional space of the first three principal components derived from a principal component analysis (PCA) (A); scree plot retaining three principal components as determined by locating the point at which the graph shows a distinct change in the slope (B); the distribution of estimated kinship values follows a normal distribution (turquoise curve) (top left) and the pairwise kinship values depicted in increasing tones of red with the clustering tree outside the matrix (C). (PPTX) [file pone.0310390.s008.pptx]
